# Frailty Index associates with *GRIN2B* in two representative samples from the United States and the United Kingdom

**DOI:** 10.1371/journal.pone.0207824

**Published:** 2018-11-26

**Authors:** Krisztina Mekli, Adam Stevens, Alan D. Marshall, Thalida E. Arpawong, Drystan F. Phillips, Gindo Tampubolon, Jinkook Lee, Carol A. Prescott, James Y. Nazroo, Neil Pendleton

**Affiliations:** 1 Cathie Marsh Institute for Social Research, The University of Manchester, Manchester, United Kingdom; 2 Division of Developmental Biology and Medicine, The University of Manchester, Manchester, United Kingdom; 3 School of Social and Political Science, The University of Edinburgh, Edinburgh, United Kingdom; 4 Department of Psychology, Dornsife College of Letters, Arts and Sciences, University of Southern California, Los Angeles, CA, United States of America; 5 Dornsife Center for Economic and Social Research, University of Southern California, Los Angeles, CA, United States of America; 6 RAND Corporation, Santa Monica, CA, United States of America; 7 Institute for Social Change, The University of Manchester, Manchester, United Kingdom; 8 Davis School of Gerontology, University of Southern California, Los Angeles, CA, United States of America; 9 Division of Neuroscience and Experimental Psychology, The University of Manchester, Manchester, United Kingdom; University of South Carolina School of Medicine, UNITED STATES

## Abstract

The concept of frailty has been used in the clinical and research field for more than two decades. It is usually described as a clinical state of heightened vulnerability to poor resolution of homeostasis after a stressor event, which thereby increases the risk of adverse outcomes, including falls, delirium, disability and mortality. Here we report the results of the first genome-wide association scan and comparative gene ontology analyses where we aimed to identify genes and pathways associated with the deficit model of frailty. We used a discovery-replication design with two independent, nationally representative samples of older adults. The square-root transformed Frailty Index (FI) was the outcome variable, and age and sex were included as covariates. We report one hit exceeding genome-wide significance: the rs6765037 A allele was significantly associated with a decrease in the square-root transformed FI score in the Discovery sample (beta = -0.01958, p = 2.14E-08), without confirmation in the Replication sample. We also report a nominal replication: the rs7134291 A allele was significantly associated with a decrease in the square-root transformed FI score (Discovery sample: beta = -0.01021, p = 1.85E-06, Replication sample: beta = -0.005013, p = 0.03433). These hits represent the *KBTBD12* and the *GRIN2B* genes, respectively. Comparative gene ontology analysis identified the pathways ‘Neuropathic pain signalling in dorsal horn neurons’ and the ‘GPCR-Mediated Nutrient Sensing in Enteroendocrine Cells’, exceeding the p = 0.01 significance in both samples, although this result does not survive correction for multiple testing. Considering the crucial role of *GRIN2B* in brain development, synaptic plasticity and cognition, this gene appears to be a potential candidate to play a role in frailty. In conclusion, we conducted genome-wide association scan and pathway analyses and have identified genes and pathways with potential roles in frailty. However, frailty is a complex condition. Therefore, further research is required to confirm our results and more thoroughly identify relevant biological mechanisms.

## Introduction

Frailty is a state of vulnerability to poor resolution of homeostasis after a stressor event and is a consequence of cumulative decline in many physiological systems over a lifetime [1]. From a clinical perspective, frailty is important because it constitutes a condition of greater risk for adverse health outcomes, such as falls, compromises in mobility, and independence, hospitalization, disability, and death [2].

Research has established that frailty associates with older age, female gender, functional dependence and chronic disease [3]. However, the pathophysiological mechanisms behind frailty are not clear. Studies with a biological focus have identified inflammation [4,5] and hormonal [6,7] dysregulation as important correlates of frailty. Other frailty-associated biomarkers include insulin growth factor, vitamin D, sirtuins, glycoproteins and cystatin C [8]. Hypothesis-driven candidate gene studies have shown associations between frailty and genes involved in inflammatory pathways [9, 10], insulin pathway, cortisol system and apoptosis [8]. These studies highlight the importance of certain genes, but fail to provide a wider picture of the multiple systems involved in frailty. Adequately powered genome-wide association scan studies supplemented with systems biology analyses can fulfil this aim by identifying multiple biological pathways with a potential role in the trait.

Several frailty assessment tools have been proposed, often using different conceptual models of frailty. The deficit model, adds together several impairments and conditions to create a Frailty Index (FI), on the grounds that the more deficits a person has, the more likely that person is to be frail. The FI can easily be constructed using information that is readily available in most health surveys [11] and it performs very well in predicting mortality compared to other frailty measures [12].

Here we report the results of a hypothesis-free genome-wide association scan (GWAS) study of frailty. We used a discovery-replication design with two independent community representative samples of older adults, the Health and Retirement Study (HRS) in the US and the English Longitudinal Study of Ageing (ELSA) in the UK. To ensure maximum replicability we used the similarly constructed FI [11] as outcome variable and a nearly identical genotyping platform in the two samples. We supplemented our study with comparative gene ontology analysis to identify key biological systems in frailty. To our knowledge this is the first study investigating the genetics of frailty in a context of systems biology. We report on genes and pathways with potential roles in frailty. Our results implicate new potential mechanisms to previously reported ones, such as inflammatory pathways and hormonal dysregulations. We expect that our results will contribute towards the better understanding of pathophysiological mechanisms behind frailty and potentiate the search for possible interventions.

## Material and methods

### Sample

We used the discovery and replication sample design. The Discovery sample was drawn from wave 9 (2008) of the Health and Retirement Study (HRS), a nationally representative sample of households of older Americans in the United States (https://hrs.isr.umich.edu/). We included participants with relatively homologous ancestry, defined by falling within 1 standard deviation of all self-identified Whites for Eigenvectors 1 and 2 for the principal component analysis and within 1 SD for fraction of heterozygotes for autosomal SNPs (http://hrsonline.isr.umich.edu/). After excluding participants with sex discrepancy, relatedness and chromosomal anomalies, we had 8539 individuals with genotype data. All participants provided written consent, and ethical approval was granted by the University of Michigan Institutional Review Board.

The Replication sample was the English Longitudinal Study of Ageing in the United Kingdom (ELSA, http://www.elsa-project.ac.uk/), a nationally representative cohort of individuals living in England aged 50 and older [13]. In this sample we included 5251 individuals who were interviewed in wave 2 (2004). Participants with non-White ethnicity or sex discrepancy were excluded. All participants provided written consent and ethical approval was granted by the London Multi-Centre Research Ethics Committee.

The investigation was carried out in accordance with the latest version of the Declaration of Helsinki.

### Genetic data

The genetic data was obtained by using Illumina's Human Omni2.5-Quad BeadChip (Illumina, San Diego, CA, USA). For the Discovery sample genotyping was performed by the NIH Center for Inherited Disease Research (Johns Hopkins University, Baltimore, MD, USA) using HumanOmni2.5-4v1 platform. For the Replication sample the University College London Genomics (London, UK) performed the genotyping on HumanOmni2.5-8v1. Initial quality control on the genetic data was performed by the data holder.

During the analysis, SNPs with minor allele frequency below 5% in the Discovery sample and below 1% in the Replication sample were also excluded, yielding 1,215,858 and 1,490,612 SNPs entering the analysis for the Discovery and Replication samples, respectively.

### Phenotypic measures

A Frailty Index (FI) was created following guidance in the literature [11]. The aim was to use a well-accepted measure which yields sufficient sample size in the genetic association analyses. Briefly, the FI counts health-related problems (deficits) in a range of domains (activities of daily living, cognitive function, falls and fractures, joint replacement, vision, hearing, chronic diseases, cardiovascular diseases, depression). The number of deficits was 45 in the Discovery sample and only individuals with non-missing values for at least 25 of them were included. In the Replication sample the number of deficits was 62, with the minimum requirement of 30 non-missing values.

Details of the development of the FI measures can be found in S1 Table.

### Statistical analysis

Phenotypic measures were developed using Stata12 software (Stata Corporation, http://www.stata.com/).

To normalise the negatively skewed distribution of the FI we performed square root transformation.

We performed linear regression analysis using the square-root transformed FI with sex and age as covariates. Regression analyses were performed using the PLINK software [14, 15].

To avoid spurious association results arising from unadjusted population substructure we used the first four Eigenvectors as covariates in the Discovery sample. The Replication sample contained only white individuals and literature indicates only modest population stratification in the British population [16]; therefore we did not include Eigenvectors in this part of the analysis.

Genomic inflation factor was calculated by R (http://cran.us.r-project.org/).

Genetic results annotation was performed by the Ensembl Variant Effect Predictor, Assembly: GRCh38.p5 [17].

We set the nominal replication criteria as p< = 0.05 and the same direction of effect in the Replication sample.

### Comparative gene ontology analysis methods

Biological pathways associated with SNPs were determined using a right-sided Fisher’s exact test (Ingenuity Pathways Analysis [IPA], Qiagen Inc, https://www.qiagenbioinformatics.com/products/ingenuity-pathway-analysis/).

## Results

### Demographic and phenotypic results

Table 1 shows that there were more females than males in both samples. Compared with the Replication sample, the Discovery sample’s mean age and mean FI were significantly higher (t-test p<0.0001). The distribution of the FI measure in the Discovery and Replication sample can be found in S2 Table.

**Table 1:** Sample characteristics.

		Discovery sample (HRS)	Replication sample (ELSA)
males (%)females (%)		3546 (41.53) 4993 (58.47)	2397 (45.67) 2851 (54.33)
Frailty Index (FI) measure	available	n = 8232	n = 5248
	mean FI (SE)	0.205 (0.0014)	0.169 (0.0015)
	mean age (SE)	69.4 (0.113)	65.9 (0.130)

### Genetic association results

We report one genome-wide significant hit. The rs6765037 A allele significantly associated with a decrease in the FI score in the Discovery sample (beta = -0.01958, p = 2.14E-08), although this was not confirmed in the Replication sample (beta = -0.005026, p = 0.2041).

We report 31 other associations below the suggestive (p<0.0001) level, spanning 10 regions in the genome. These SNPs are in high linkage disequilibrium (LD) with each other within one region, therefore there are fewer independent signals. For example, among the 7 SNPs identified on chromosome 14, the lowest LD was 0.917296 between rs73301475 and rs17093546, suggesting only one independent signal in this region. The 32 associations exceeding the suggestive level can be found in S3 Table.

We report one successful nominal replication between the Discovery and Replication samples. The rs7134291 A allele significantly associated with a decrease in the FI score (Discovery sample: beta = -0.01021, p = 1.85E-06, Replication sample: beta = -0.005013, p = 0.03433).

The genomic inflation factors were 1.033 for the Discovery and 1.001 for the Replication sample. They did not indicate serious population stratification in the samples. We also provide Manhattan and QQ plots to visualise our results, which can be found in S4 Table.

### Comparative gene ontology analysis results

**Fig 1:**
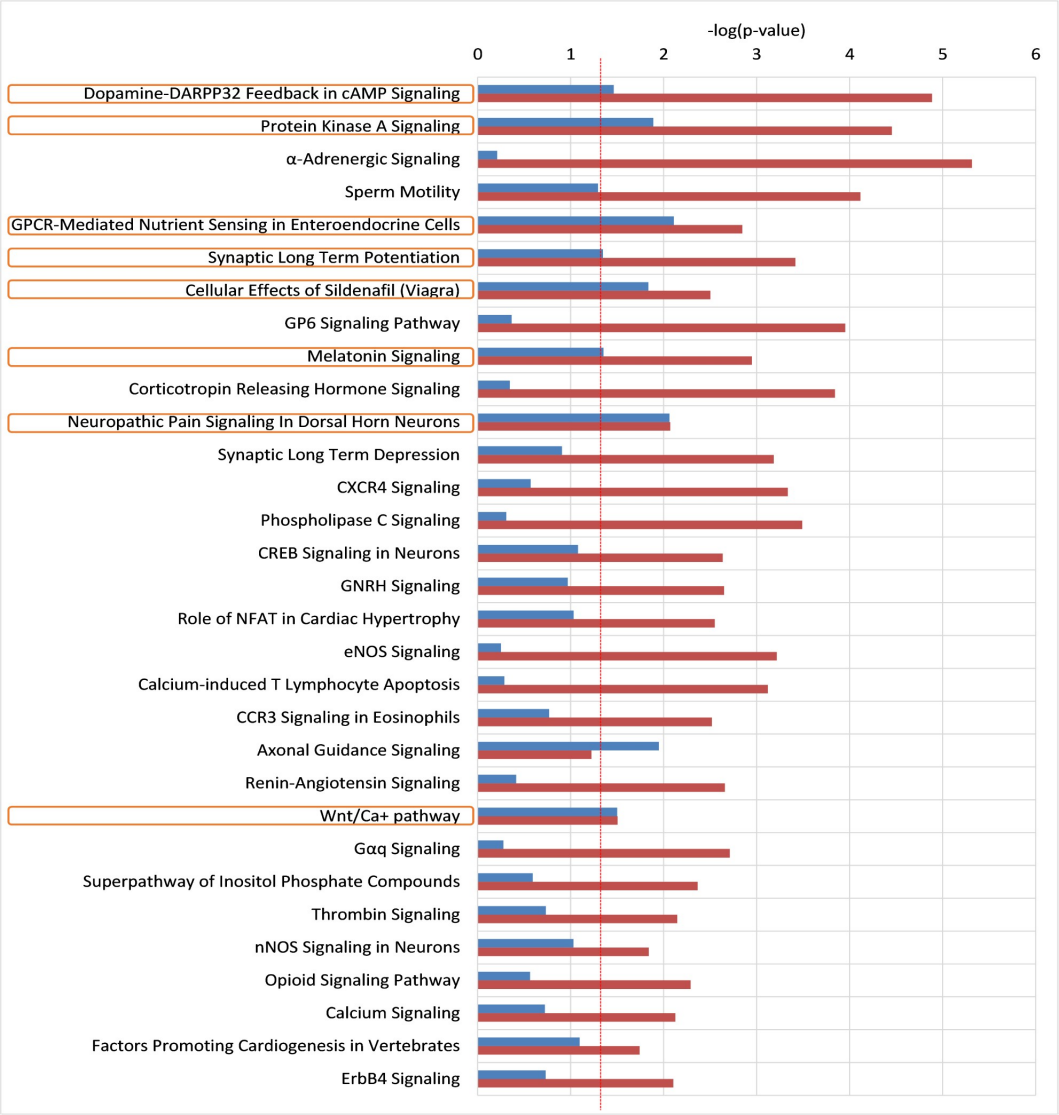
Results of the comparative gene ontology analysis. Eight pathways were shown to be significantly (p<0.05) represented in the groups of SNPs derived from both studies, they are marked with orange boxes. Horizontal red line indicates the p = 0.05 significance level. Significance levels on a negative log scale are indicated with blue (Discovery sample) and red (Replication sample) bars.

Eight pathways were shown to be significantly (p<0.05) represented in the groups of SNPs derived from both analyses. Of the pathways identified, the ‘Neuropathic pain signalling in dorsal horn neurons’ and the ‘GPCR-Mediated Nutrient Sensing in Enteroendocrine Cells’ achieved p’s<0.01 in both samples (Fig 1). None of these results survive Bonferroni-correction for multiple testing.

## Discussion

To our knowledge, our study is the first genome-wide association study on the deficit model of frailty. Our aim was to use a hypothesis-free approach to identify genes and pathways in order to understand pathophysiological mechanisms behind frailty. We used two independent, representative samples from the US and the UK. We chose a widely accepted frailty measure, the Frailty Index [11] as the outcome variable.

Our most significant association was between the rs6765037 A allele and a decrease in the FI score. This SNP located in an intergenic region of chromosome 3, close to the 5’ region of the *KBTBD12* (Kelch repeat and BTB Domain containing 12) gene. This gene is not well characterised in the literature. According to the Human Protein Atlas, higher RNA expression was found in the heart muscle and in the skeletal muscle [18]. We did not find any past associations in the literature indicating the role of *KBTBD12* or rs6765037 in any phenotype relevant to frailty.

The nominally replicated SNP, rs7134291 is located on chromosome 12, within the first intron of the *GRIN2B* (Glutamate Ionotropic Receptor NMDA Type Subunit 2B) gene. *N*-methyl-d-aspartate receptors (NMDARs) are a family of ionotropic glutamate receptors that mediate a slow Ca^2+^ permeable component of excitatory synaptic transmission in the central nervous system. NMDARs are a tetrameric assembly with the *GRIN2B* encoding the glutamate-binding NR2B subunit [19]. NR2B together with the NR1 and NR2A subunits are expressed in the human cerebral cortex during the second trimester of gestation, a period of intense neurogenesis and synaptogenesis, suggesting involvement of NMDARs in the maturation of human cortical neurons and in early synapse formation [20]. Overexpression of NR2B in the forebrain (cortex and hippocampus) has been shown to lead to increased learning and memory abilities in behavioural tasks in young adult mice [21], as well as in ageing mice [22]. In humans, variants in the *GRIN2B* gene have been associated with impaired cognitive phenotypes such as intellectual disability [23], and developmental delay [24, 25].

NMDA receptors are activated by L-glutamate and glycine. Binding of these co-agonists must take place for the ion channel to open fully. Extracellular Mg^2+^ blocks the ion channel absent membrane depolarization, which requires prior activation of AMPA and kainate receptors. This process is thought to underlie the contribution of NMDARs to synaptic plasticity and long-term potentiation (LTP) [26]. Long-term potentiation in the cortex is a long-lasting highly localised increase in synaptic strength and a molecular substrate for memory and learning [27]. Loss of synaptic plasticity is a characteristic of Alzheimer’s disease (AD) and the molecular mechanism possibly involves the NMDARs. Beta-amyloid (Aβ) oligomers, one of the toxic protein species believed to be etiologically related to AD, impair LTP and enhance long-term depression [28]. Aβ reduces glutamatergic transmission and inhibits synaptic plasticity through regulating the number of NMDARs. [29]. Application of Aβ has been found to promote endocytosis of NMDARs in cortical neurons, and neurons from a genetic mouse model of AD expressed reduced amount of surface NMDARs [30]. These observations have been supplemented with genetic studies that found associations between *GRIN2B* variants and AD [31, 32]. One of the studies, conducted in a Northern Chinese sample found that the frequency of the rs3764028 C allele was higher in AD cases than in controls, even in APOE ε4-negative cases. This is an upstream SNP, for which a Luciferase reporter assay showed 35–40% lower promoter activity for the rs3764028 C allele compared with the A allele. This indicates that the major C allele might decrease the transcriptional activity of *GRIN2B*. Rs3764028 was not present in our dataset, with the closest SNP being rs12368476, 1077 bp away. The rs12368476 A allele associated with a decrease in the FI in both samples (HRS: beta = -0.003893, p = 0.045, ELSA: beta = -0.005127, p = 0.017). Our nominally replicated SNP, rs12368476, is 7819 bp away from rs12368476 with an r^2^ of 0.4478 between them. It is possible, that one or more SNPs in the upstream region of *GRIN2B* decrease the promoter activity of the gene and hence the association with FI and AD, via decreased synaptic plasticity. The involvement of synaptic plasticity in frailty is further supported by the fact that the ‘Synaptic long-term potentiation’ pathway was among the eight pathways exceeding the p = 0.05 threshold significance in our comparative gene ontology analysis.

Considering the crucial role of *GRIN2B* in brain development, synaptic plasticity and cognition, this gene appears to be a plausible candidate to play a role in frailty.

It is possible that the found association is driven by cognitive deficit, which is included in our frailty model. The association may also be indirect, manifesting through other deficits, which are associated with cognition, such as sensory impairment [33] or depression [34]. These deficits are part of the FI, which includes self-reported eyesight and hearing and 8 questions from the Center for Epidemiologic Studies Depression Scale questionnaire (CESD; [35]). However, frailty is defined as a disorder of several inter-related physiological systems [1] and our operationalisation of the FI in both the Discovery and Replication samples included markers of this full range of systems; therefore *GRIN2B*’s role in cognition is unlikely to be a comprehensive explanation for its association with such a complex phenotype.

Indeed, in the GWAS literature, *GRIN2B* has been linked to various other traits. Relevant to our study are IL2 secretion [36], time to major incident event [37] and FSH levels in women [38].

Aging is characterized by raised levels of proinflammatory cytokines such as interleukin-1 (IL-1), interleukin-6 (IL-6) and tumour necrosis factor (TNF) as well as reduced IL-2 levels, reflecting a low-grade chronic systemic proinflammatory state, termed *inflammaging*. As a result, the immune system declines in responsiveness and efficiency resulting in greater susceptibility to age-related diseases and frailty. In this context, the association of a *GRIN2B* variant, rs2268118, with interleukin-2 (IL-2) secretion to vaccinia virus stimulation in smallpox vaccine recipients [36] is noteworthy. Although it appears a plausible candidate, we have not found studies investigating associations between frailty or any related phenotype and IL-2.

Literature suggests that *GRIN2B* may also have a role in human longevity. A GWAS study on time to event (defined by major incident events, such as myocardial infarction, heart failure, stroke, dementia, hip fracture, or cancer) or death, as an alternative phenotype for healthy aging, found that another genetic variant in *GRIN2B*, rs4764043, was associated with increased risk of event [37]. This is relevant to our study, as these items are part of the FI measure (except for death) and they increase the prediction of mortality compared to an FI without these items [39]. The results of the Walter study implies *GRIN2B*’s involvement of the pathophysiology of these diseases through yet unknown mechanisms, and in this way would increase risk of frailty.

A third variant, rs6488619, has been associated with follicle-stimulating hormone (FSH) levels in Caucasian women [38]. Higher levels of FSH were associated with higher FI in a study of 3219 men [7]. In this case the association may be indirect, as higher FSH levels could be markers of testicular dysfunction, which itself is a sensitive marker of homeostatic disruption and poor overall health status in older men.

Of these SNPs, rs4764043 and rs6488619 were included in our GWAS, but neither associated with the FI (results not shown).

Our nominally replicated SNP, rs7134291, has shown some association with triglyceride levels in the literature, although this did not reach genome-wide significance [40]. We found no association between rs7134291 and any lipid biomarker in our study (results not shown).

Taking these results together, *GRIN2B* appears to be a plausible candidate in the pathophysiology of frailty, but these results also indicate the involvement of other mechanisms.

Comparative gene ontology analysis identified two pathways exceeding the 1.0E-02 threshold in both samples. The first pathway is the ‘Neuropathic pain signalling in dorsal horn neurons’ with the NMDARs having an important role in it. Neuropathic pain is generally defined as a chronic pain state resulting from peripheral or central nerve injury, or both, and is likely to be due to long-term plastic changes along the nociceptive pathway. The spinal cord dorsal horn is the first relay station of nociceptive information from periphery to the brain and NMDAs are expressed here abundantly [41, 42]. Activation of postsynaptic NMDA receptors not only participates in glutamatergic sensory synaptic transmission in normal conditions, but more importantly is also involved in the spinal dorsal horn in pathological pain conditions induced by tissue inflammation or nerve injury [43]. All NMDAR subunits are expressed in the spinal dorsal horn; however, NR2B exhibits the largest expression among NR2 subunits [41]. There has been evidence in the literature of an association between pain and the Frailty Index. A study using our Replication sample found that pain status is predictive of incident and worsening frailty [44], as did a prior study of men with chronic widespread pain [45].

The second pathway is the ‘GPCR-Mediated Nutrient Sensing in Enteroendocrine Cells’. Food intake is detected by the chemical senses of taste and smell and subsequently by chemosensory cells in the gastrointestinal tract that link the composition of ingested foods to feedback circuits controlling gut motility/secretion, appetite, and peripheral nutrient disposal. A number of G-protein-coupled receptors (GPCRs) have been identified as potential ‘‘sensors” of luminal nutrients. These receptors stimulate the release of gut peptides via coupling to Gα_S_, which elevates intracellular cAMP, and Gα_q_, which then results in Ca^2+^ mobilization and Protein Kinase C activation. Inhibitory influences are exerted by signalling through Gα_i_ pathways that decrease cAMP levels [46]. As anorexia in aging, defined as a loss of appetite and/or reduced food intake, affects a significant number of elderly people and is far more prevalent among frail individuals [47], this pathway may play a role in frailty.

Our study has considerable strengths. We used large sample sizes in two independent cohorts. The very similarly constructed phenotypic measures and the nearly identical genotyping platform between the two cohorts ensures maximum replicability. Our samples consist of community dwelling individuals, who are not hospitalised or institutionalised, therefore frailty is not overrepresented. The FI distribution is similar to that previously reported in the literature [11] S2 Table.

Our study suffers from some limitations. First, the FI measure contained 45 deficits in the Discovery sample and 62 deficits in the Replication sample. However, the missing 17 items in the Discovery sample were distributed across each health domain covered by the Index, so this is unlikely to pose a serious problem. Moreover, an Index with 30–40 variables has been shown to be sufficiently accurate for predicting adverse outcomes [11]. Second, frailty may be influenced by environmental factors, such as nutrition or access to health care, which we did not account for. The latter might be especially important as the health care system is rather different between the US and the UK. Also, the use of samples from different countries may result in cohort effects. FI is a complex phenotype, and therefore is likely to be influenced by many genes with small effects, our study could be underpowered to find associations for less common, but possibly important variants, despite our best efforts. This insufficient power may explain the lack of confirmation of previously reported correlates of frailty, such as members of the inflammatory pathway (*IL-1β*, *IL-6* and *TNF*) and apoptosis (rs129968 in *CREBBP* and rs3769827 in *CASP8*) [8] in our study. In future studies larger sample sizes will be required to detect these small effects.

Finally, we note that the two pathways exceeding the 1.0E-02 threshold in the gene ontology analysis did not survive adjustment for multiple testing, despite their potential biological relevance. This may be due to insufficient power; therefore additional genetic association and experimental studies are needed to verify these potentially important pathways.

In conclusion, our study’s main finding implicates the importance of the *GRIN2B* and a pathway with NR2B playing a potential role using two different analytical techniques in two independent samples. However, as frailty is a complex condition, further research is required to corroborate our findings and reveal further pathways associated with frailty.

## Supporting information

S1 Table Phenotypes. Lists of deficits for the Frailty Index.
(XLSX)

S2 Table Graphs. Distribution of the phenotypic variable.
(XLSX)

S3 Table GenAssoc. The most significant results of the genome-wide association scan.
(XLSX)

S4 Table Plots. QQ and Manhattan plots of the GWAS.
(XLSX)
